# Improving casemix systems by integrating functioning information: a systematic literature review and call for expert input

**DOI:** 10.1186/1472-6963-14-S2-P57

**Published:** 2014-07-07

**Authors:** Maren Hopfe, Gerold Stucki, Mirjam Brach, Birgit Prodinger

**Affiliations:** 1 Swiss Paraplegic Research, Nottwil, Switzerland; 2 Department of Health Sciences & Health Policy, University of Lucerne, Lucerne, Switzerland

## Background

An aging population and growing number of people with chronic health conditions, alongside resource constraints in health systems, require innovative and integrative approaches to improve systems’ efficiency while meeting the needs of individuals and populations. Health information is the foundation for evidence-based decision making within and across all levels of health systems. Understood comprehensively from the perspective of human functioning, health information encompasses bio-medical or disease-specific components as well as information on how health plays out in daily life. This comprehensive perspective demonstrates the complexity of health and is needed to facilitate an integrated care approach across settings and individuals’ life span. Though disease-specific and functioning information are conceptually complimentary, on an operational level current reimbursement systems, especially casemix systems, still rely predominantly on information about the disease. There is, however, increasing evidence that the mere diagnosis fails to predict, e.g. length of stay, discharge destination, and service costs. The need to adapt current casemix systems to the joint use of disease and functioning information is recognized in order to account for the complexity of health. Health systems rely on evidence on the added value of functioning information to decide whether to adapt their casemix systems. The objectives of this paper are i) to provide preliminary findings of a systematic literature review that aims to identify the added value of integrating functioning information into casemix systems, and ii) to raise challenges in integrating functioning information into casemix systems for discussion with experts.

## Materials and methods

We are currently performing a systematic literature review using standard literature databases (PubMed, EMBASE, CINAHL, Sociological Abstracts, JSTOR and EconLit) and hand-search of reference lists. English-language papers describing empirical studies that include a comparative component in their study design, account for functioning information in casemix systems, and those published in peer-reviewed international journals will be included. Based on the preliminary findings of the review, issues and benefits of integrating functioning information will be raised for discussion.

## Results and conclusions

This review examines the evidence for the added value of comprehensive health information in health systems to inform decisions toward adapting casemix systems to account for the complexity of health. Any decision related to reimbursement is interrelated with service delivery, policy and governance in a health system. Hence, it requires the involvement of various stakeholders in decisions on adapting current casemix systems. This paper calls for expert input on how to proceed with this research agenda.

